# Role of Epidural Analgesia within an ERAS Program after Laparoscopic Colorectal Surgery: A Review and Meta-Analysis of Randomised Controlled Studies

**DOI:** 10.1155/2016/7543684

**Published:** 2016-08-24

**Authors:** Giuseppe Borzellino, Nader Kamal Francis, Olivier Chapuis, Evguenia Krastinova, Valérie Dyevre, Michele Genna

**Affiliations:** ^1^Department of Surgery, University Hospital of Verona, Piazzale A. Stefani 1, 37126 Verona, Italy; ^2^Yeovil District Hospital and University of Bath, District Hospital NHS Foundation Trust, Taunton, UK; ^3^Clinique Chirurgicale Val d'Or, Paris, France; ^4^Clinical Research Platform, URC-CRC, Saint Antoine Hospital, APHP, Paris, France

## Abstract

*Introduction.* Epidural analgesia has been a cornerstone of any ERAS program for open colorectal surgery. With the improvements in anesthetic and analgesic techniques as well as the introduction of the laparoscopy for colorectal resection, the role of epidural analgesia has been questioned. The aim of the review was to assess through a meta-analysis the impact of epidural analgesia compared to other analgesic techniques for colorectal laparoscopic surgery within an ERAS program.* Methods.* Literature research was performed on PubMed, Embase, and the Cochrane Library. All randomised clinical trials that reported data on hospital stay, postoperative complications, and readmissions rates within an ERAS program with and without an epidural analgesia after a colorectal laparoscopic resection were included.* Results.* Five randomised clinical trials were selected and a total of 168 patients submitted to epidural analgesia were compared to 163 patients treated by an alternative analgesic technique. Pooled data show a longer hospital stay in the epidural group with a mean difference of 1.07 (95% CI 0.06–2.08) without any significant differences in postoperative complications and readmissions rates.* Conclusion.* Epidural analgesia does not seem to offer any additional clinical benefits to patients undergoing laparoscopic colorectal surgery within an ERAS program.

## 1. Introduction

Since the early reporting of fast-track surgery, epidural analgesia has been considered a cornerstone of such a recovery program [1]. It is widely perceived that epidural analgesia alters the stress response after surgery, improves pain control, and consequently reduces cardiovascular and infectious complications [2]. However, a recent meta-analysis on the role of epidural analgesia within an enhanced recovery after surgery (ERAS) program in open surgery has reported better pain control but no reduction in postoperative stay and in complications rates with epidural analgesia compared to other analgesic techniques [3].

In colorectal surgery, the laparoscopic technique has been widely adopted as a safe alternative to open surgery due to its beneficial effects on the reduction of postoperative pain and postoperative ileus, which can shorten the length of hospital stay [4]. Laparoscopy is supposed to induce a weaker neuroendocrine response to the surgical trauma to a level that could permit one to not include the epidural analgesia within the ERAS program. The finding that epidural analgesia, both within and outside any ERAS program, does not affect the length of hospital stay after laparoscopic colorectal surgery may support this hypothesis [5]. Moreover a recent review has reported that an ERAS program may reduce hospital stay after laparoscopic colorectal surgery while the laparoscopic approach may reduce morbidity when compared to the open approach within an ERAS program [6].

These results question the role of epidural analgesia in laparoscopic colorectal surgery within an ERAS program. However, reporting of results on epidural analgesia is heterogeneous across different studies and no single study provides a level of evidence high enough to draw definitive conclusions on the exact role of epidural analgesia in laparoscopic colorectal surgery within an ERAS program [7–13].

This study aims to systematically review the literature reporting on the impact of epidural analgesia with laparoscopic colorectal surgery when delivered within an ERAS program and to conduct a meta-analysis of the results of randomised controlled studies.

## 2. Methods

The meta-analysis was performed according to the PRISMA statement for reporting reviews and meta-analysis [14]. The present review was not registered on the PROSPERO database for meta-analysis.

### 2.1. Literature Research

Literature research was conducted on PubMed, Embase, and the Cochrane Library with the following term strategy: (((((((epidural) OR eras) OR enhanced recovery) OR fast track) OR rehabilitation)) AND ((((colon surgery) OR colorectal surgery)) AND laparoscop^∗^)) AND ((randomized) OR randomised); and it was completed by hand search of the selected studies.

### 2.2. Eligibility Criteria

Studies published between January 2000 and January 2016 were included to promote the capture of all papers published since ERAS was most widely implemented in clinical practice. The title and the abstract were checked for a first selection of relevant studies; papers were selected for full reading and a second selection was performed through the review of the entire article. Papers were selected for inclusion in the meta-analysis only if they reported a randomised controlled trial comparing epidural analgesia with other analgesic techniques within an ERAS program in laparoscopic colorectal surgery, and they reported the data related to at least one of the following: hospital stay, postoperative complications rate, and readmissions rate. Papers that compared epidural and spinal analgesia were not included. No language restriction was applied. Case studies, nonrandomised comparative studies, and studies which did not report an ERAS program were excluded. Abstracts and conference proceedings were excluded because of the probability of incomplete data.

### 2.3. Data Extraction and Methodological Quality

Data were extracted and crosschecked from the papers by two independent authors. Since patients are not usually discharged until full recovery, the postoperative hospital stay was chosen as the main efficacy outcome for the meta-analysis. For assessment of the effect of the analgesic method of the recovery program on safety, postoperative complications and readmission rates were also extracted for the pooled analysis. A sensitivity analysis was performed by analysing the estimated results with both the random and the fixed effect model, by dividing the studies according to the alternative analgesic technique, by eliminating discordant studies according to the design of the studies. The following data were collected for a secondary analysis: pain scores, functional outcomes such as time to first flatus, time to first bowel movement, time to resolution of postoperative nausea and vomiting (PONV), or rate of patients with PONV and time to oral diet intake. All the authors have been contacted and have been asked to provide lacking data and to provide data as mean values and their SD for continuous variables, when necessary. Two independent reviewers reviewed the studies. Methodological quality of the randomised studies was assessed using the Cochrane collaborations tool for assessing risk of bias [15].

### 2.4. Statistical Analysis

When continuous variables were given as a median and range or interquartile range (IQR), transformation in mean and standard deviation was performed by the described methods of Hozo et al. [16] and Wan et al. [17]. The weighted mean difference (WMD) and the 95% confidence interval were calculated for the pooled estimation of continuous variables, and the relative risk (RR) and the 95% confidence interval were calculated for the pooled estimation of dichotomous variables.

Since selected studies report a comparison between epidural analgesia and other different analgesic techniques, heterogeneity of results was expected; the random effect model was therefore adopted and data pooled using the DerSimonian Laird method [18]. Heterogeneity was estimated with the *χ* ^2^ test and the *I* ^2^ statistic. Heterogeneity was excluded when *I* ^2^ was less than 30% [19, 20]. The meta-analysis was conducted using the Review Manager (RevMan) computer program Version 5.3 [21].

## 3. Results

**Figure 1:**
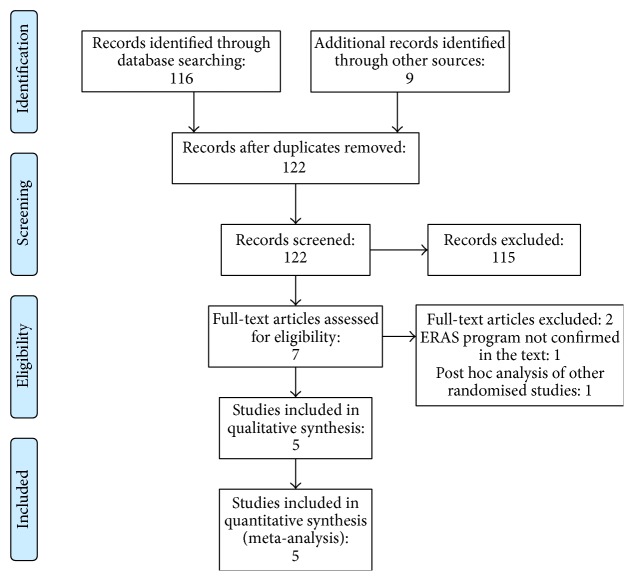
PRISMA diagram of study selection.

A PRISMA diagram of selected studies is summarised in Figure 1. The search strategy identified 125 abstracts, from which 3 duplicates were removed, leaving 122 papers. A further 115 were excluded by review of the title and/or abstract because they did not meet the eligibility criteria, leaving a total of 7 papers selected for a full paper reading. One study was eliminated because the ERAS programme was not described or even mentioned in the text, and another one was eliminated because it reported a post hoc analysis of another trial, leaving 5 randomised clinical trials included in this study for data extraction and meta-analysis. This included a total of 168 patients treated by epidural analgesia and 163 by another analgesic technique.

**Figure 2:**
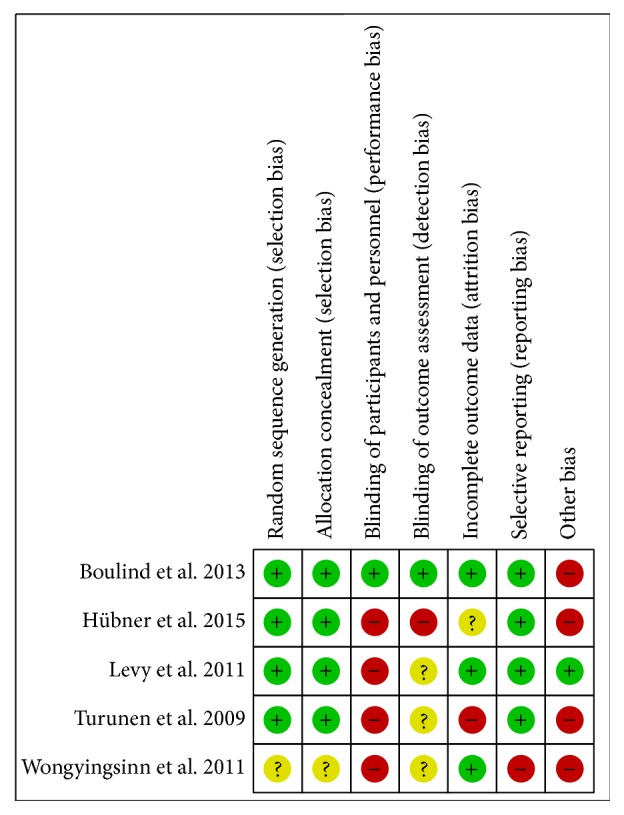
Risk of bias assessment.

The characteristics and details of the included studies are summarised in Table 1. The bias assessment is reported in Figure 2. All studies were randomised but among them only one reported blinding; it was however a pilot study and no hypothesis or no sample size calculation was provided [10]. Intention to treat analysis was not performed in all studies [7–9, 11] because of the noninclusion of some patients after randomisation. The alternative analgesic techniques to which epidural analgesia was compared were heterogeneous among studies. In one study epidural analgesia was evaluated as an add-on to an IV analgesic regimen [7], one study compared epidural analgesia with spinal analgesia and IV PCA [8], another one study compared epidural analgesia to intravenous patient controlled analgesia (IV PCA) [11] within a slightly different protocol, one study compared epidural analgesia with IV lidocaine [9] and another one with a wound infusion catheter [10]. The items of the ERAS program were clearly listed in three studies [7–9] while in two studies the items were not listed; the ERAS program the study referred to was just mentioned or cited [10, 11].

### 3.1. Quantitative Analysis

**Figure 3:**
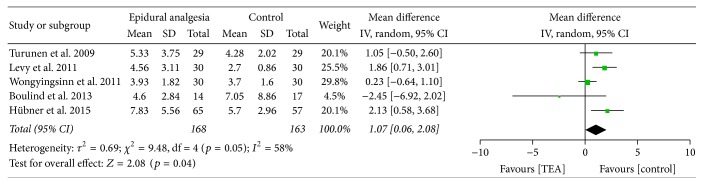
Hospital stay.

The length of hospital stay was reported in all five studies and was statistically longer in the epidural groups than in alternative analgesic technique groups with a WMD of 1.07 (95% CI 0.06–2.08) as shown in Figure 3. However a significant heterogeneity was found with *I* ^2^ being 58%.

**Figure 4:**
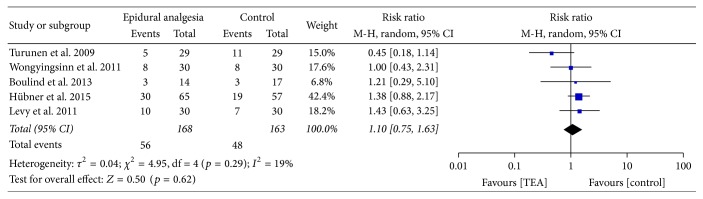
Postoperative complications.

Postoperative complications were reported in all the five studies; no difference was found between the epidural regimen and other analgesic techniques with RR of 1.1 (95% CI 0.75–1.63) as shown in Figure 4. No significant heterogeneity was found.

**Figure 5:**
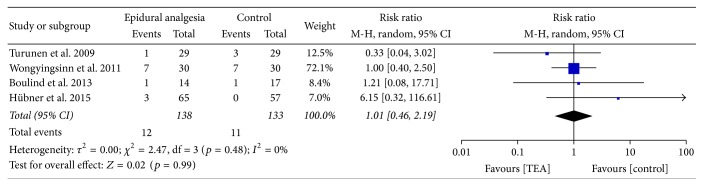
Readmissions.

Rates of readmission were reported in four studies; no difference was found between the epidural regimen and other analgesic techniques with RR of 1.01 (95% CI 0.46–2.19) as shown in Figure 5. No heterogeneity was found.

### 3.2. Sensitivity Analysis

No different results were found by estimating pooled data with the fixed effect model. The estimated WMD of hospital stay was 0.98 (95% CI 0.4–1.56), the estimated RR of postoperative complications rate was 1.11 (95% CI 0.81–1.53), and the estimated RR of readmissions was 1.08 (95% CI 0.51–2.27).

By restricting the data estimations to studies using the same alternative analgesic technique [8, 11], a higher difference of postoperative stay was found between epidural analgesia and IV PCA. By pooling data from the studies by Levy et al. [8] and Hübner et al. [11], the estimated WMD of postoperative stay was 1.96 (95% CI 1.03–2.88) favouring IV PCA. No significantly different results were found with estimated RR of postoperative complications, which was 1.39 (95% CI 0.94–2.07). The estimated RR of readmission could not be calculated since Levy et al. did not specify the rate of readmissions.

By eliminating the study by Wongyingsinn et al., which included low rectal surgery with ileostomy, and that by Boulind et al., which was a pilot study on blinding feasibility, a higher difference in postoperative stay was found. The estimated WMD was 1.72 (95% CI 0.92–2.51), while no difference was found in postoperative complications with an estimated RR of 1.04 (95% CI 0.55–1.99) and in readmission with an estimated RR of 1.21 (95% CI 0.07–21.65).

### 3.3. Secondary Outcomes Analysis

A qualitative analysis was performed for the secondary outcomes because of limited homogeneity in the reporting of data.

The patients' pain score was assessed by a visual analogue scale or by verbal rate scale in all studies but reporting and results were heterogeneous among the studies. The study by Boulind et al. reported better pain control with epidural at D0 but not at D2; mean pain scores with SD were 0.6 (0.4) with epidural and 3.4 (2) with wound infusion catheter on D0, 2.4 (2) with epidural, and 2.6 (2.2) with wound infusion catheter on D1, while pain scores were 3.2 (3.1) with epidural and 2.4 (2.9) with wound infusion catheter on D2 but no statistical evaluation was reported [10]. The study by Levy et al. [8] found that pain score was significantly better in the epidural group at D0 both at rest and on movement, reporting a median value with IQR of VAS on movement of 4,5 (IQR 2,8–6) with epidural and 7 (IQR 6–8) with PCA (*p* < 0.001); but no significant differences on pain scores were found at D1 and D2 between epidural and PCA. In the study by Turunen et al. [7] rates of patients with significant pain (VAS ≥ 3) were compared, finding no difference at rest (*p* = 0.171) but a higher number of patients with pain at mobilisation in the nonepidural group (*p* = 0.001). The study by Wongyingsinn et al. [9] reported a median value and IQR for colon and rectal surgery separately. No differences were reported in the pain scores at rest, on coughing, and on walking after colonic surgery, while a better pain control was registered with epidural analgesia after rectal surgery at rest with pain scores of 0 (0–2) versus 3 (1.5–3) (*p* = 0.023) at D1 and 0 (0–2) versus 3 (1–3) (*p* = 0.008) at D2. The study by Hübner et al. [11] found no significant differences between epidural analgesia and PCA; the authors reported detailed data on a figure.

Three studies reported data on the time to first flatus. No differences were found between epidural analgesia and alternative analgesic techniques in all the studies. Turunen et al. [7] reported a median value of 1 (1–4) day both in the epidural group and in the control group (*p* = 0.219). Levy et al. [8] reported median values of 1.5 (IQR 0.9–2.2) days in the epidural group and 1.6 (IQR 1.2–2) days in the PCA group (*p* not reported). Wongyingsinn et al. [9] reported in the colon surgery group without ileostomy a mean value of 24 hours (95% CI 19–29) in the epidural group and 27 hours (95% CI 22–32) in the IV lidocaine group (*p* = 0.38).

Three studies reported data on the time to first bowel movement. No differences were found between epidural analgesia and alternative analgesic techniques in all the studies. Turunen et al. [7] reported a median value of 2 (1–9) days in the epidural group and 2 (1–7) days in the control group (*p* = 0.56). Levy et al. [8] reported median values of 3.1 (IQR 2.25–4.75) days in the epidural group and 4 (IQR 2.43–4.55) days in the PCA group (*p* = 0.346). Wongyingsinn et al. [9] reported in the primary anastomosis group without ileostomy a mean value of 44 hours (95% CI 35–52) in the epidural group and 43 hours (95% CI 34–52) in the IV lidocaine group (*p* = 0.887).

Two studies reported data on PONV with discordant results. Levy et al. [8] reported a longer duration of nausea and vomiting in the epidural group with median values, respectively, of 1.7 (IQR 0.95–4.45) versus 0.55 (IQR 0–1.55) days (*p* = 0.006) for nausea and 1 (IQR 0–2.15) versus 0 (IQR 0–0.25) days (*p* = 0.008) for vomiting. Wongyingsinn et al. [9] reported no different rates of patients having suffered of nausea with rates of 57% in the epidural groups versus 37% in the IV lidocaine group (*p* = 0.438) and of vomiting with rates of 60% in the epidural group versus 40% in the IV lidocaine group (*p* = 0.791).

Similar results were reported for time to oral intake. In the study by Levy et al. [8], the time to oral intake was longer in the epidural group with a median of 2 (IQR 1.29–4.6) versus 1.43 (IQR 0.8–2.3) days (*p* = 0.02). No difference was found in the study by Wongyingsinn et al. [9] with a mean time of oral intake of 35 hours (95% CI 22–51) in the epidural group versus 38 hours (95% CI 22–46) in the IV lidocaine group (*p* = 0.894).

## 4. Discussion

The present meta-analysis summarised the reporting of five randomised controlled trials evaluating the impact of epidural analgesia following laparoscopic colorectal surgery within an ERAS program. This review found that there was a significantly longer hospital stay, without any significant difference in postoperative complications and readmissions rates in the group of patients managed with an epidural analgesia compared to other analgesic techniques. The mean difference in hospital stay may be considered as fairly relevant, since patients with epidural analgesia were discharged 1 day later than patients treated with other analgesic techniques.

All included studies in the present meta-analysis are randomised and in all studies [7–12] the random sequence generation and allocation concealment were reported and were unclear just in one study, giving strength to the results of the meta-analysis. However, although blinding is very important when evaluating pain control, only the study by Boulind et al. [10] was blind. It was furthermore a pilot study on the feasibility of blinding in randomised studies on epidural analgesia; it was not designed to explore a clinical hypothesis and no sample size calculation was performed. Finally the designs of the included studies were not homogeneous; different analgesic alternative techniques had been investigated; the explored hypothesis and the main measured outcome were different for each of the included studies.

A significant heterogeneity test and a 58% inconsistency index were found with pooled data on hospital stay. By eliminating one pilot study and one study that included low rectal surgery with ileostomy, a more significant difference was found, up to 1.72 days with no heterogeneity, giving more strength to the result of a longer hospital stay with epidural analgesia compared to other analgesic techniques. The length of postoperative hospital stay reflects the quality of the postoperative course since a confident degree of autonomy needs to be raised before discharge. Discharge may however be influenced by the subjectivity of any medical decisions, by socioeconomic factors and expectance of patients, which are independent of the investigation but all are supposed not to influence comparisons in a randomised setting. In the present meta-analysis, on one hand the length of hospital stay was significantly shorter in the alternative analgesic technique group, but no differences have been found in most of the single criteria generally used to discharge. Although some of them have been reviewed only through a qualitative analysis, no differences were found in postoperative complications rate, time to flatus, and time to first bowel movement. The study by Levy et al. [8] reported a longer period of PONV and time to oral diet intake in the epidural group. Considering the number of included patients in the study by Levy et al., the shorter duration of PONV compared to time to discharge, there is low probability that the study has influenced results of the meta-analysis on postoperative length of stay. Based on these considerations and on the absence of homogeneous criteria for patient discharge in the selected papers, a possible bias on the length of hospital stay cannot be excluded.

When considering the pooled data on safety, the power of the meta-analysis should be interpreted with some degree of caution given the lack of difference on postoperative complications and readmissions rates. Nevertheless, considering the number of patients included in the pooled analysis and the absence of significant heterogeneity, the results may be considered being at low risk of underestimation of any difference between the effects of epidural and alternative analgesic techniques on postoperative complications and readmissions rates.

Literature data on the role of epidural analgesia in colorectal surgery are still controversial. According to the more recent guidelines, positioning of an epidural catheter is strongly recommended in open colon surgery but could be probably avoided in laparoscopic colorectal surgery with, however, a low grade of recommendation [22, 23]. The present review supports the latter recommendation based on reviewing the randomised controlled trials in this area. This is, to our knowledge, the first meta-analysis on the effect of epidural analgesia within an ERAS program in laparoscopic colorectal surgery.

Epidural analgesia has been advocated for postoperative pain control, for the management of the neuroendocrine response, and for its effects on postoperative stay [1, 2]. A retrospective study [24] and two randomised studies [25, 26] both reported that epidural analgesia outside an ERAS program improved pain control in laparoscopic colorectal surgery. A randomised study on laparoscopic sigmoid resection did not report pain control improvement with epidural analgesia, but only 20 patients were included [27]. The results of the present review on pain control are heterogeneous. Furthermore, it must be underlined that both epidural and other analgesic techniques were provided as an add-on to IV antalgic treatment regimen in all the studies; risk of confounding or attenuating the effect of the randomised analgesic techniques cannot therefore be excluded. The advantages of an ERAS program including epidural analgesia in laparoscopic colorectal surgery have been reported, both by comparison to open surgery within an ERAS program [28] and by comparison of an ERAS program versus conventional care within laparoscopic colorectal surgery [29, 30]. The question however remains on whether epidural analgesia offers any additional benefits, through neuroendocrine blockage, to laparoscopic surgery within an ERAS program. The role of other items in an ERAS program other than epidural analgesia should be investigated, since no high level of evidence-based studies on this issue has been found [22, 23]. The effects of epidural analgesia on the length of hospital stay outside an ERAS program have been reported in two studies. A randomised study reported that epidural analgesia did not affect the length of hospital stay in laparoscopic colorectal surgery [25] and a retrospective study based on a trial database found that laparoscopy was an independent predictive factor of early recovery, but epidural analgesia was not [31].

Side effects and feasibility of the epidural analgesia were not reported in the present review; however they should be taken into account when deciding to recommend or not the epidural analgesia. The side effects of epidural analgesia have not been reported to be significantly higher than with other analgesic techniques [6], but epidural analgesia may be the cause of rare but serious complications such as epidural hematoma and neurological sequels [4]. Moreover, the placement of a catheter for the epidural analgesia is not always successful; rates of failure from 13% to 32% have been reported in major abdominal surgery [32].

The meta-analysis has some limits. The review has included studies where the sample size was not assessed for the length of hospital stay, which is the main outcome of the meta-analysis, except that of Levy et al. [8]. Only one included study was blinded, and it was a pilot study with no exploration of any clinical hypothesis [10]. Design of the studies and the alternative analgesic techniques compared to epidural analgesia were heterogeneous among the included trials, and most important for the clinical implication the items of the ERAS programs were not well described in all the studies and were not homogenous.

The present meta-analysis confirmed that epidural analgesia might not offer any additional clinical benefits to patients undergoing laparoscopic colorectal surgery within an ERAS program. However, the results of this review are limited by the number of studies and should be interpreted with some degree of caution. A further large multicentre randomised controlled trial appears necessary to definitively recommend not performing an epidural analgesia within an ERAS program in colorectal surgery.

## Figures and Tables

**Table 1:** Summary of studies' characteristics.

Authors	Studies	Interventions	Alternative to epidural	Number of patients with epidural/alternative analgesia	Epidural protocol	Alternative protocol	Hypothesis	Main measured outcome
Turunen et al. 2009 [7]	Open RCT	Laparoscopic sigmoidectomy for diverticular diseases	Epidural on add-on to opioids	29/29	Daily doses: Ketoprofen 100 mg × 3 Paracetamol 1 gr × 4 If needed Oxycodone 0,05 mg/kg IV or 0,15 mg/kg IM + Epidural infusion Ropivacaina 2 mg/mL at 4 to 10 mL/h for 2 days	Daily doses: Ketoprofen 100 mg × 3 Paracetamol 1 gr × 4 If needed Oxycodone 0,05 mg/kg IV or 0,15 mg/kg IM	Epidural analgesia reduced use of opioids and therefore advanced postoperative outcomes	Postoperative IV Oxycodone consumption

Levy et al. 2011 [8]	Subgroup analysis within a triple comparison in open RCT; patients with stoma excluded	Laparoscopic colorectal surgery for benign and malignant diseases	PCA	30/30	Diclofenac 50 mg × 3 Paracetamol 1 gr × 4 In case of allergy: Tramadol 50–100 mg or Morphine 2.5–10 mg + Epidural infusion Bupivacaine 0.15% Fentanyl 0.0002% 4–8 mL/h for 2 days	Diclofenac 50 mg × 3 Paracetamol 1 gr × 4 In case of allergy: Tramadol 50–100 mg or Morphine 2.5–10 mg + PCA pump Morphine maximum dose 20 mg every 4 hours	Exploration of the effects of different analgesic regimens on postoperative outcomes	Length of postoperative stay

Wongyingsinn et al. 2011 [9]	Open RCT Included patients with ileostomy	Laparoscopic colorectal surgery	IV Lidocaine infusion	30/30	Acetaminophen 1 gr × 4 Naproxen 500 mg × 2 + Epidural infusion Bupivacaine 0.1% Morphine 0.02 mg/mL for 2 days	Acetaminophen 1 gr × 4 Naproxen 500 mg × 2 + IV catheter Lidocaine 2 mg/kg per hour As recue PCA IV catheter 1-2 mg Morphine every 7 minutes	Comparison of epidural analgesia and IV Lidocaine infusion	Return to bowel function

Boulind et al. 2013 [10]	Pilot study of blinded RCT	Laparoscopic colorectal surgery for benign and malignant diseases	Wound infusion catheter (WIC)	14/17	Epidural infusion Doses not reported	Wound catheter infusion Doses not reported	Feasibility of a large RCT comparing epidural analgesia and WIC	No main primary outcome

Hübner et al. 2015 [11]	Open RCT	Elective colorectal surgery	PCA	65/57	Metamizole 500 mg × 4 Paracetamol 1 gr × 4 + Epidural infusion Bupivacaine 0.1% Fentanyl 2 *μ*g/mL Adrenaline 2 *μ*g/mL + 3 mL bolus every 40 minutes	Metamizole 500 mg × 4 Paracetamol 1 gr × 4 + PCA IV catheter Fentanyl 1 mg/h + 1 mL bolus every 5 minutes up to 40 mg/4 h	Superiority of epidural over PCA	Mean reduction of medical recovery time

RCT: randomised clinical trial.

WIC: wound infusion catheter.

PCA: patient controlled analgesia.

IV: intravenous.

IM: intramuscular.
